# Transforming growth factor‐β enhances Rho‐kinase activity and contraction in airway smooth muscle via the nucleotide exchange factor ARHGEF1

**DOI:** 10.1113/JP275033

**Published:** 2017-11-23

**Authors:** Yasin Shaifta, Charles E. MacKay, Nneka Irechukwu, Katie A. O'Brien, David B. Wright, Jeremy P. T. Ward, Greg A. Knock

**Affiliations:** ^1^ Division of Asthma, Allergy and Lung Biology, Faculty of Life Sciences and Medicine King's College London London SE1 1UL UK

**Keywords:** airway smooth muscle, TGF‐β, Rho‐kinase

## Abstract

**Key points:**

- Transforming growth‐factor‐β (TGF‐β) and RhoA/Rho‐kinase are independently implicated in the airway hyper‐responsiveness associated with asthma, but how these proteins interact is not fully understood.
- We examined the effects of pre‐treatment with TGF‐β on expression and activity of RhoA, Rho‐kinase and ARHGEF1, an activator of RhoA, as well as on bradykinin‐induced contraction, in airway smooth muscle.
- TGF‐β enhanced bradykinin‐induced RhoA translocation, Rho‐kinase‐dependent phosphorylation and contraction, but partially suppressed bradykinin‐induced RhoA activity (RhoA‐GTP content).
- TGF‐β enhanced the expression of ARHGEF1, while a small interfering RNA against ARHGEF1 and a RhoGEF inhibitor prevented the effects of TGF‐β on RhoA and Rho‐kinase activity and contraction, respectively.
- ARHGEF1 expression was also enhanced in airway smooth muscle from asthmatic patients and ovalbumin‐sensitized mice.
- ARHGEF1 is a key TGF‐β target gene, an important regulator of Rho‐kinase activity and therefore a potential therapeutic target for the treatment of asthmatic airway hyper‐responsiveness.

**Abstract:**

Transforming growth factor‐β (TGF‐β), RhoA/Rho‐kinase and Src‐family kinases (SrcFK) have independently been implicated in airway hyper‐responsiveness, but how they interact to regulate airway smooth muscle contractility is not fully understood. We found that TGF‐β pre‐treatment enhanced acute contractile responses to bradykinin (BK) in isolated rat bronchioles, and inhibitors of RhoGEFs (Y16) and Rho‐kinase (Y27632), but not the SrcFK inhibitor PP2, prevented this enhancement. In cultured human airway smooth muscle cells (hASMCs), TGF‐β pre‐treatment enhanced the protein expression of the Rho guanine nucleotide exchange factor ARHGEF1, MLC_20_, MYPT‐1 and the actin‐severing protein cofilin, but not of RhoA, ROCK2 or c‐Src. In hASMCs, acute treatment with BK triggered subcellular translocation of ARHGEF1 and RhoA and enhanced auto‐phosphorylation of SrcFK and phosphorylation of MYPT1 and MLC_20_, but induced de‐phosphorylation of cofilin. TGF‐β pre‐treatment amplified the effects of BK on RhoA translocation and MYPT1/MLC_20_ phosphorylation, but suppressed the effects of BK on RhoA‐GTP content, SrcFK auto‐phosphorylation and cofilin de‐phosphorylation. In hASMCs, an ARHGEF1 small interfering RNA suppressed the effects of BK and TGF‐β on RhoA‐GTP content, RhoA translocation and MYPT1 and MLC_20_ phosphorylation, but minimally influenced the effects of TGF‐β on cofilin expression and phosphorylation. ARHGEF1 expression was also enhanced in ASMCs of asthmatic patients and in lungs of ovalbumin‐sensitized mice. Our data indicate that TGF‐β enhances BK‐induced contraction, RhoA translocation and Rho‐kinase activity in airway smooth muscle largely via ARHGEF1, but independently of SrcFK and total RhoA‐GTP content. A role for smooth muscle ARHGEF1 in asthmatic airway hyper‐responsiveness is worthy of further investigation.

## Key points

- Transforming growth‐factor‐β (TGF‐β) and RhoA/Rho‐kinase are independently implicated in the airway hyper‐responsiveness associated with asthma, but how these proteins interact is not fully understood.
- We examined the effects of pre‐treatment with TGF‐β on expression and activity of RhoA, Rho‐kinase and ARHGEF1, an activator of RhoA, as well as on bradykinin‐induced contraction, in airway smooth muscle.
- TGF‐β enhanced bradykinin‐induced RhoA translocation, Rho‐kinase‐dependent phosphorylation and contraction, but partially suppressed bradykinin‐induced RhoA activity (RhoA‐GTP content).
- TGF‐β enhanced the expression of ARHGEF1, while a small interfering RNA against ARHGEF1 and a RhoGEF inhibitor prevented the effects of TGF‐β on RhoA and Rho‐kinase activity and contraction, respectively.
- ARHGEF1 expression was also enhanced in airway smooth muscle from asthmatic patients and ovalbumin‐sensitized mice.
- ARHGEF1 is a key TGF‐β target gene, an important regulator of Rho‐kinase activity and therefore a potential therapeutic target for the treatment of asthmatic airway hyper‐responsiveness.

## Introduction

In healthy subjects, airway smooth muscle (ASM) tone and airflow resistance are low, while in subjects with allergic asthma, exposure to allergens sensitizes airways to constrictor stimuli, resulting in inappropriate episodic ASM constriction (airway hyper‐responsiveness, AHR) and long‐term remodelling (Doeing & Solway, 2013). These smooth muscle phenotypic changes are triggered by inflammatory mediators, produced in the airways in response to allergen sensitization. One of these mediators, transforming growth factor‐beta (TGF‐β), is produced at higher basal levels in asthmatic airways and is increased several‐fold after allergen challenge (Redington *et al*. 1997; Torrego *et al*. 2007) up to levels that correlate with the severity of AHR and remodelling (Matsunaga *et al*. 2006). In connective tissue, TGF‐β induces contractile protein expression and stress fibre assembly (Vardouli *et al*. 2005; Sandbo *et al*. 2011; Ji *et al*. 2014), while in ASM, it can influence both proliferation and hypertrophy (Halwani *et al*. 2011) and induce hyper‐responsiveness to bronchoconstrictors (Goldsmith *et al*. 2006; Oenema *et al*. 2012).

Smooth muscle cross‐bridge cycling requires MLC_20_ (myosin light‐chain 20‐kDa subunit) phosphorylation, which is in turn dependent on the balance between activity of Ca^2+^‐induced myosin light‐chain kinase and myosin light‐chain phosphatase (MLCP). The latter is inhibited by phosphorylation of myosin phosphatase targeting subunit‐1 (MYPT1) by Rho‐kinase, an effector of the monomeric G‐protein RhoA (Somlyo & Somlyo, 2003). Thus, TGF‐β could mediate enhanced ASM responsiveness to bronchoconstrictors by enhancing expression or activity of RhoA/Rho‐kinase, their downstream targets or their upstream regulators. Among the upstream regulators are the Rho‐specific guanine nucleotide exchange factors (RhoGEFs), a large family of regulatory proteins that activate members of the Rho family of small G‐proteins by catalysing the exchange of bound GDP for bound GTP (Bos *et al*. 2007). Two of these, GEF‐H1 and Net1, are up‐regulated by TGF‐β in epithelia, thus enhancing RhoA‐dependent F‐actin expression/cell migration and epithelial‐to‐mesenchymal transition, respectively (Tsapara *et al*. 2010; Papadimitriou *et al*. 2012). Another RhoGEF, ARHGEF1 (p115‐RhoGEF, Lsc), not previously linked with TGF‐β, has been implicated in the T‐cell dysfunction associated with allergic asthma, and in the contractile effects of angiotensin II on hypertensive vascular smooth muscle (Brown *et al*. 2007; Guilluy *et al*. 2010). The role of ARHGEF1 in ASM contraction remains to be determined.

We have recently confirmed that bradykinin (BK)‐induced MLC_20_ phosphorylation and contraction in healthy rat airways and cultured human ASM was dependent on Rho‐kinase activity, which was also partly mediated via activation of Src‐family kinases (SrcFK) (Shaifta *et al*. 2015). Both Rho‐kinase and SrcFK have been suggested as potential therapeutic targets in asthma, both having being implicated in experimental AHR (Chiba *et al*. 2005; Schaafsma *et al*. 2006; Katsumoto *et al*. 2013). Smooth muscle contraction is also influenced by factors that regulate actin cytoskeleton re‐organization and contractile apparatus assembly; in several cell types this is also reportedly mediated by RhoA/Rho‐kinase, in part via inhibitory phosphorylation of cofilin (Arber *et al*. 1998; Vardouli *et al*. 2005; Leduc *et al*. 2016). Conversely, it has been suggested that, particularly in ASM, cofilin has an alternative role in promoting actin polymerization, perhaps independently of Rho‐kinase (Zhao *et al*. 2008; Zhang *et al*. 2010). In this study we therefore examined the long‐term effects of TGF‐β on BK‐induced ASM contraction, as well as on protein expression and activity of ARHGEF1, SrcFK, RhoA/Rho‐kinase MYPT1/MLC_20_ and cofilin.

## Methods

### Ethical approval

All studies with rat tissues were performed *in vitro* after animals were killed by an intra‐peritoneal sodium pentobarbital injection. Ovalbumin (OVA) sensitization and lung function measurement in mice was performed in accordance with The Animals (Scientific procedures) Act (1986) and local ethical approval from King's College London. Donation of human tissue was obtained following written informed consent and with the approval of the South East London Research Ethics Committee, REC reference number: 10/H0804/66. All clinical procedures conformed to the standards set by the latest Declaration of Helsinki.

### Animal tissue

Investigations of airway reactivity in healthy animals were conducted in isolated small intralobar bronchioles obtained from male Wistar rats (∼250 g). Lungs were removed immediately after killing by lethal injection (pentobarbital, i.p.). Bronchioles (∼1 mm in diameter, ∼2 mm in length) were dissected free of surrounding parenchyma and placed in cold physiological saline solution (PSS, composition in mm: 118 NaCl; 24 NaHCO_3_; 1 MgSO_4_; 4 KCl; 5.56 glucose; 0.435 NaH_2_PO_4_; 1.8 CaCl_2_, pH 7.4). Lung tissue was also obtained from a mouse model of airway hyper‐responsiveness. 129SVJ/Black Swiss mice were immunized four times at 7 day intervals, with OVA (30 μg ml^−1^ i.p., OVA‐treated), or vehicle [Al_2_(OH)_3_, sham‐treated]. They were then challenged with aerosolized OVA (30 μg ml^−1^, 2 × 25 min per day for 4 days). Airway hyper‐responsiveness in OVA‐treated mice was confirmed by greater methacholine‐induced reduction in airway dynamic compliance (Cdyn) compared to sham‐treated (*P* < 0.01) and greater sensitivity to methacholine compared to sham‐treated (Cdyn half‐maximal provocation concentration (PC50) reduced by 50%, *P* < 0.001).

### Human tissue and cell culture

Human airway smooth muscle cells (hASMCs) were obtained from healthy volunteers (*n* = 11; 7 female, 4 male; age range 22–53 years) or volunteers with moderate asthma (*n* = 3; all male; age range 44–53 years), using deep endobronchial biopsy. Healthy volunteers were selected based on a life‐long absence of respiratory symptoms, and lung functions within normal limits. Moderate asthmatics were selected based on the presence of typical symptoms over at least 2 years, a minimum of 12% reversibility, forced expiratory volume in 1 sec (FEV1) <80% of predicted for age, and/or a bronchoconstrictor response to a methacholine challenge of <8 mg ml^−1^. All asthmatic subjects were on a combined therapy of short acting beta‐agonist and inhaled corticosteroids (fluticasone propionate <500 μg daily or equivalent). Patients currently receiving leukotriene receptor antagonists, smokers and pregnant/lactating females were excluded.

ASM bundles were bathed in Dulbecco's modified Eagle medium (DMEM) containing the following supplements: 10% fetal bovine serum, l‐glutamine (2 mm), sodium pyruvate (1 mm), non‐essential amino acids and amphotericin B (2 μg ml^−1^) and dissected free of adjacent connective tissue and epithelia. They were then subjected to enzymatic digestion in nominally Ca^2+^‐free Hepes buffer containing: 5.56 mm glucose, 2 mg ml^−1^ collagenase Type XI, 1 mg ml^−1^ papaine, 1 mg ml^−1^ trypsin inhibitor and 1 mm DTT. Cells were then dispersed, incubated in DMEM (plus supplements) at 37°C, pH 7.4, and typically used for experiments at passage 4–9. hASMCs were seeded onto 60 mm dishes, grown to confluence and serum starved for 7 days in DMEM without FBS, supplemented with l‐glutamine (2 mm), sodium pyruvate (1 mm), non‐essential amino acids (1:100), amphotericin B (1.5 μg ml^−1^), gentamicin (50 μg ml^−1^), insulin‐transferrin‐selenium (1:100), 100 μm ascorbate and bovine serum albumin (BSA) (1%). This prolonged serum starvation and media/supplement combination has previously been shown to restore and preserve a contractile phenotype in ASM (Halayko *et al*. 1999; Kim *et al*. 2005). hASMCs were confirmed as smooth muscle by positive staining for smooth muscle‐specific protein markers, as described previously (Shaifta *et al*. 2015).

### TGF‐β treatments

The long‐term effects of TGF‐β on tissue and cellular function and protein expression were examined by pre‐incubation of rat bronchioles or hASMCs for 18–24 h at concentrations similar to those previously found to exist in the airways of asthmatic patients (Redington *et al*. 1997; Torrego *et al*. 2007). Bronchioles were incubated for 18 h at 37°C with 30 ng ml^−1^ TGF‐β serum‐free Ham's F12 medium with supplements and 0.1% BSA, or in Ham's F12 medium and supplements/BSA alone (Halayko *et al*. 1999; Kim *et al*. 2005). Following this incubation, bronchioles were transferred to PSS for subsequent tension measurement by wire myography, in the absence of TGF‐β. Treatment of hASMCs with 10 ng ml^−1^ TGF‐β for 24 h was initiated at day 6 of serum starvation, followed by washout with serum‐free media (without TGF‐β) for a further 24 h, prior to acute treatments with pharmacological agents and harvesting.

### Tension measurement by wire myography

Bronchioles were mounted on a Mulvany–Halpern small vessel wire myograph (www.DMT.dk), and bathed in PSS gassed with 95% air/5% CO_2_ at 37°C, for the measurement of isometric tension. Bronchioles were stretched incrementally, and alternately exposed to PSS containing 80 mm [K^+^] (equimolar substitution for Na^+^, KPSS) to determine the point on the length–tension curve at which optimum active tension development was achieved, as described previously (Moir *et al*. 2003). Viability of bronchioles for tension experiments was confirmed by active tension generation of at least 2 mN in response to the last exposure to KPSS. Mean internal diameter after stretching was typically in the range 300–800 μm.

### RhoA‐EmGFP/ARHGEF1‐EmGFP cloning

RhoA and ARHGEF1 cDNAs were separately cloned into pcDNA6.2/C‐EmGFP/TOPO to produce C‐terminal Emerald green fluorescent protein (EmGFP) fusion vectors (Invitrogen Life Technologies, UK). Oligonucleotide primers were designed in accordance with the manual and using sequences from the GenBank database (RhoA accession no. BC061732; ARHGEF1 accession no. BC091218) as follows: RhoA (forward): GTTATGGCTGCCATCAGGAAGAAACTGG‐3′, RhoA (reverse): 5′‐CAAGATGAGGCACCCCGACT‐3′, ARHGEF1 (forward): 5′‐GAGATGGGAGAAGTCGCCGGAGGGGC‐3′, ARHGEF1 (reverse): 5′‐TGAAAGGCCTGTCTGAGCAGAGCGC‐3′. Clones were checked for the correct orientation using restriction endonuclease digestion and gel electrophoresis. These were then sent for sequencing (Source BioScience, Nottingham, UK) and selection.

### siRNA design and transfection

The human ARHGEF1 small interfering RNA (siRNA) was designed as described previously (Knock *et al*. 2008; Shaifta *et al*. 2015). The 19 nucleotide target sequence (position 2057–2075, GenBank accession no. NM_199002) was synthesized into 64–65 mer oligonucleotides with *Bam*HI/*Hind*III overhangs (Sigma, Poole, UK) and cloned into the expression vector pSilencer 3.0‐H1 (Ambion Inc., Austin, TX, USA). All clones were purified (EndoFree Plasmid Maxi Kit, Qiagen, Valencia, CA, USA) and sequenced (Source BioScience). As a negative control, another siRNA was prepared using a scrambled mRNA sequence. Transfection was carried out in detached confluent hASMCs using the Basic Nucleofector Kit for Primary Mammalian Smooth Muscle Cells and a nucleofector device (Nucleofector Technology ‐ Lonza AG, Basel, Switzerland). The medium was changed the next day and cells were grown for a further 48 h in DMEM plus supplements, prior to serum starvation and TGF‐β treatment, as described above. Transfection efficiency was >90%, determined separately using pmaxGFP (green fluorescent protein expressing vector) provided in the kit and confirmed by fluorescence microscopy. The efficiency of ARHGEF1 knockdown was confirmed by western blot (see Fig. 3). If designed correctly, siRNA can be a highly specific tool for targeted gene knockdown (Semizarov *et al*. 2003). To minimize any non‐specific targeting, candidate siRNAs presenting more than 16 contiguous nucleotides of sequence identity with another mRNA were discarded (Birmingham *et al*. 2007). A BLAST alignment (NCBI, Bethesda, MD, USA) of the 19 nucleotide ARHGEF1 siRNA sequence showed only 5 contiguous nucleotide homology to MLC_20_ (GenBank accession no. S69022), RhoA (GenBank accession no. BC061732) and cofilin (GenBank accession no. D00682), only 6 contiguous nucleotide homology to PDZ‐RhoGEF (ARHGEF11, GenBank accession no. BC057394), only 7 contiguous nucleotide homology to LARG (ARHGEF12, GenBank accession no. NM01513) and ROCK2 (GenBank accession no. NM004850), and only 9 contiguous nucleotide homology to MYPT1 (GenBank accession no. D87930).

### Rho‐GTP content measurement by Rhotekin asssay

After treatment, cells were harvested as described above using MLB lysis buffer made up according to The Rho Assay Reagent protocol (Millipore‐Merck, Billerica, MA, USA). The lysates were then cleared of insoluble cell debris by centrifugation, a small amount taken to determine protein concentration and the remainder immediately snap frozen and stored at –80°C. In the pull‐down assay the collected samples were quick‐thawed and 250–500 μg of protein was mixed with 20 μg of The Rho Assay Reagent slurry (A GST‐tagged fusion protein, corresponding to residues 7–89 of mouse rhotekin Rho‐binding domain bound to glutathione‐agarose) and incubated for 45 min at 4°C with gentle agitation using a roller mixer. As a positive control, an extra untreated sample was pre‐incubated with GTPγS for 30 min at 30°C prior to mixing with The Rho Assay Reagent slurry. After 45 min, the mixture was centrifuged at 4°C and the supernatant discarded followed by three washes with ice cold MLB, again centrifuging each time and discarding the supernatant. After the final wash, 40 μl of 2× Laemmli reducing sample buffer containing 50 mm DTT (to improve release of RhoA from the beads) was added and the mixture was boiled at 95°C for 5 min, followed by cooling and storage at −20°C or below. The final supernatant and agarose pellet were mixed before being subjected to SDS‐PAGE and western blot, as described below.

### RhoA‐EmGFP/ARHGEF1‐EmGFP translocation imaging and quantification

Coverslips containing serum‐starved hASMCs were mounted on to a Zeiss Axiovert 200 microscope and cells were visualized using a BD CARV II Confocal Imager under ×40 magnification, bathed in PSS/5% CO_2_ at 37°C. When illuminated with UV/blue light (∼390 nm), successfully transfected cells were identified by bright green fluorescence. RhoA‐EmGFP/ARHGEF1‐EmGFP translocation was stimulated by acute application of 1 μm BK. Images were captured by MetaFluor v.7 software (Molecular Devices, Sunnyvale, CA, USA). This typically involved brief (65 ms) shutter openings, once every 2 s during stimulation, but less frequently during rest periods. Regional and temporal changes in fluorescence, indicating movement of the EmGFP‐tagged protein in response to drug treatments, were quantified using ImageJ software (rsb.info.nih.gov). Briefly, a line was drawn across each region of interest centred around spots or patches on the cell periphery. For each region, a single value of difference in fluorescence between the ‘spot’ peak intensity and nearby background was determined. Each region of interest was measured before and during exposure to BK in the same cell. Two or three regions of interest were analysed and averaged for each cell and BK‐induced fluorescence expressed as a percentage of control. The only criteria for exclusion of a cell from the analysis was poor EmGFP transfection, indicated by overall cellular fluorescence below a pre‐determined threshold level.

### hASMC acute treatment, harvesting and western blot

SrcFK, MYPT1, MLC_20_ and cofilin phosphorylation/de‐phosphorylation was stimulated by acute treatment with BK (1 μm, 30 s) in serum‐free DMEM at 37°C, as shown previously (Shaifta *et al*. 2015). Cells were then immediately washed twice with ice‐cold PBS (Invitrogen, Carlsbad, CA, USA) to terminate reactions and remove any residual media, followed immediately by application of cell lysis buffer (New England Biolabs, Ipswich, MA, USA) containing 1% phosphatase inhibitor cocktails 2 and 3 and 1% protease inhibitor cocktail (all Sigma). Cells were scraped into a tube, agitated continuously for 30 s, placed on ice and centrifuged at 10000 r.p.m. for 5 min. The resultant pellets were discarded and the remaining supernatant was stored at −80°C for future use.

Protein content was determined using the bicinchoninic acid assay, calibrated against BSA protein standards. Prior to gel loading for SDS‐PAGE, lysates were prepared in NuPAGE LDS Sample Buffer (Invitrogen) and protein content was adjusted so that each lane of the gel would typically contain 20 μg of protein. Samples were boiled at 95°C for 5 min before being loaded onto 4–12% NuPAGE Bis‐Tris gels (Invitrogen). Gels were run in Mops running buffer (Invitrogen) and protein was transferred to nitrocellulose membrane (GE Healthcare, Little Chalfont, UK) in transfer buffer (25 mm Tris, 192 mm glycine, 20% methanol).

Membranes were blocked with 5% skimmed milk in Tris‐buffered saline (TBS), followed by incubation with specific anti‐phospho‐protein primary antibody (typically 1:1000 dilution) in TBS with 1% skimmed milk and 0.1% Tween‐20 (TBS‐T), overnight at 4°C. This was then followed by washes in TBS‐T and the application of appropriate HRP‐conjugated secondary antibody (typically 1:5000 dilution) for 1 h at room temperature, followed by further washes in TBS‐T. ‘Phospho’ proteins were visualized with Super‐Signal West Femto chemi‐luminescent Substrate (Thermo Scientific, Waltham, MA, USA). Membranes were then stripped in Restore western blot stripping buffer (Thermo Scientific), re‐blocked and re‐incubated with corresponding ‘total’ antibody and appropriate secondary antibodies, as above. ‘Total’ proteins were visualized with either ECL plus or ECL prime (GE Healthcare). All images were captured and quantified using the ChemiDoc XRS+ gel imaging system (Bio‐Rad, Hercules, CA, USA).

Changes in protein expression were determined as a ratio of the target protein signal over signal for the ‘housekeeping’ protein GAPDH, acting as a loading control for each sample. An estimate of the amount of target protein that was phosphorylated in response to acute treatments was calculated both as a ratio of ‘phospho’ over ‘total’ signal for each protein sample, to give an indication of the fraction of each protein that is phosphorylated in response to BK, and as a ratio of ‘phospho’ over GAPDH, to reveal the influence of TGF‐β and ARHGEF1 siRNA on total phosphorylation of each protein, in addition to the acute effect of BK. The effects of treatments on these ratios were then expressed as a percentage of control (no BK, no TGF‐β, run on the same gel).

### Materials and reagents

Antibodies were obtained from: Cell Signaling [Danvers, MA, USA: anti‐phospho‐Src (tyr416), anti‐Src, anti‐phospho‐MLC (ser19), anti‐MLC, anti‐MYPT1, anti‐p115RhoGEF, anti‐RhoA, anti‐cofilin, anti‐phospho‐cofilin (ser3)], Millipore [anti‐phospho‐MYPT1 (thr696)], Sigma (anti‐rabbit IgG, anti‐mouse IgG) and Santa Cruz Biotechnology (Santa Cruz, CA, USA: anti‐ROCK2). All antibodies were used at 1:1000 dilution except for anti‐phospho‐MYPT1, anti‐rabbit IgG and anti‐mouse IgG, which were used at 1:5000. PP2 [4‐amino‐5‐(4‐chlorophenyl)‐7‐(dimethylethyl) pyrazolo[3,4‐d]pyrimidine] was obtained from Calbiochem; BK, Y16 (inhibitor of RGS‐domain containing RhoGEFs) and Y27632 (Rho‐kinase inhibitor) were obtained from Sigma. Rhotekin reagent and GTP‐γ‐S were obtained from Millipore. TGF‐β was obtained from R&D Systems (Minneapolis, MN, USA). Cell culture and western blot materials were from Cell Signaling, Invitrogen, GE Healthcare or Thermo Scientific. General reagents were from Sigma, Calbiochem or VWR (Radnor, PA, USA).

### Data analysis and statistics

All values are expressed as mean ± SEM. All statistical analysis and non‐linear regression curve fitting was performed with SigmaPlot 10. BK concentration responses were biphasic, so were fitted using a two‐site saturation model, for the characterization of a high affinity component (PD2‐1 and Max‐1) and a low affinity component (PD2‐2 and Max‐2). In contraction experiments, ‘*n*’ refers to the number of bronchioles obtained from a similar number of rats. In western blot experiments, ‘*n*’ refers to the number of cell cultures, each derived from a different human subject. In translocation experiments, ‘*n*’ refers to the number of cells, selected at random from at least three separate cell cultures, each derived from a different human subject. Statistical comparisons were by paired or un‐paired *t* test (two groups of data, single factor), one‐way, two‐way or three‐way ANOVA (multiple comparisons, single factor, two‐factor or three‐factor, respectively), with Holm‐Sidak post‐tests where appropriate, and as indicated in figure or table legends. Differences were considered significant at *P* < 0.05.

## Results

### TGF‐β enhances BK‐induced constriction via RhoGEFs and Rho‐kinase, but not SrcFK

**Figure 1:**
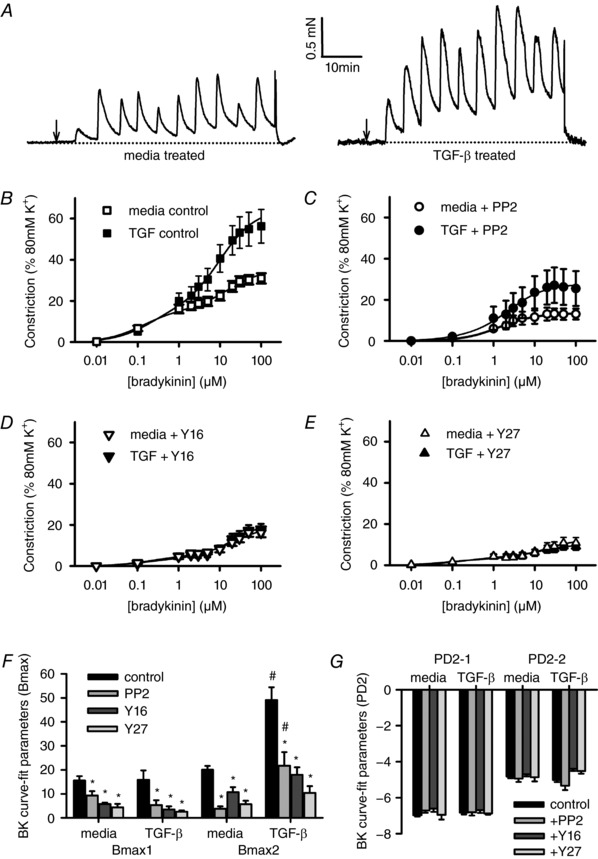
Effects of TGF‐β and SrcFK/RhoGEF/Rho‐kinase inhibition on BK‐induced contraction Measurement of isometric tension in isolated rat bronchioles mounted on the wire myograph. *A*, representative traces showing bradykinin (BK) applied cumulatively (0.01–100 μm) at 5 min intervals, after 18 h pre‐incubation in media only or in media with 30 ng ml^−1^ TGF‐β. Arrows indicate when the first dose of BK was applied. *B*–*E*, mean contraction amplitude after TGF‐β or media pre‐incubation measured at the plateau phase of each BK application, in the absence (*B*, media: *n* = 24, TGF‐β: *n* = 23) or presence of 30 μm PP2 (*C*, media: *n* = 17, TGF‐β: *n* = 15), 30 μm Y16 (*D*, media: *n* = 9, TGF‐β: *n* = 9) or 10 μm Y27632 (*E*, media: *n* = 9, TGF‐β: *n* = 9). *F* and *G*, non‐linear regression data, showing effects of TGF‐β and inhibitors on Bmax (*F*) and PD2 values (*G*). ^*^ *P* < 0.05 *vs*. control, ^#^ *P* < 0.05 *vs*. media, two‐way ANOVA.

The contractile effects of BK (0.01–100 μm) on isolated rat bronchioles were determined with or without prior exposure to TGF‐β. The BK dose response was then repeated in each bronchiole in the presence of the SrcFK inhibitor PP2 (30 μm), the inhibitor of RGS domain‐containing RhoGEFs Y16 (30 μm; Shang *et al*. 2013) or the Rho‐kinase inhibitor Y27632 (10 μm). At each dose, BK produced a biphasic contraction, incorporating a large transient which usually decayed to a near plateau within 5 min. Non‐linear regression analysis of the plateau phase at each concentration showed that the data were best fitted to a two‐site saturation model with a high and a low affinity component. In the absence of inhibitor, TGF‐β enhanced the contractile response compared to media alone (Fig. 1 *A* and *B*). Specifically, the amplitude of the low affinity component (Bmax‐2) was increased by TGF‐β (Fig. 1 *F*), while other parameters were unaltered (Fig. 1 *F* and *G*). All three inhibitors partially suppressed BK‐induced contraction, after both media and TGF‐β pre‐incubation (Fig. 1 *C*–*E*), showing significant reductions in amplitude of both high and low affinity components (Bmax‐1 and Bmax‐2). However, there remained a significant difference in Bmax2 between media and TGF‐β pre‐treated bronchioles in the presence of PP2, while in the presence of Y16 or Y27632 these differences were no longer significant. Other curve‐fit parameters were unaffected by the three inhibitors, regardless of pre‐exposure to TGF‐β or media alone (Fig. 1 *G*). TGF‐β pre‐exposure did not alter the amplitude of KPSS‐induced constriction compared to media (media only: 2.9 ± 0.4 mN, *n* = 49 *vs*. TGF‐β: 3.0 ± 0.4 mN, *n* = 49). Repeating BK dose‐responses in the absence of inhibitor (time controls), in bronchioles exposed to TGF‐β or media alone, showed no changes in any of the curve‐fit parameters (*n* = 4, data not shown).

**Figure 2:**
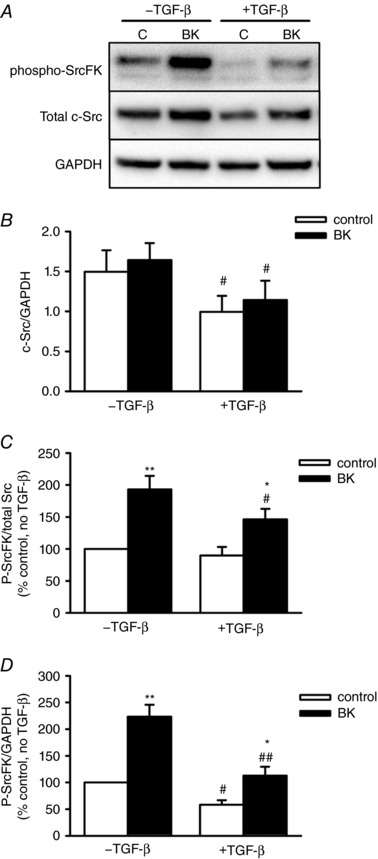
Effects of BK and TGF‐β on SrcFK expression and auto‐phosphorylation in hASMC *A*, representative blots showing effect of BK (1 μm, 30 s) with or without prior exposure to TGF‐β (10 ng ml^−1^, 24 h) on phospho‐SrcFK content, total c‐Src content and GAPDH as a loading control. *B*, data expressed as c‐Src/GAPDH show partial inhibition of c‐Src protein expression after TGF‐β pre‐incubation (^#^ *P* < 0.05 *vs*. –TGF‐β, *n* = 7). *C* and *D*, SrcFK phosphorylation is enhanced by BK (^*^ *P* < 0.05, ^**^ *P* < 0.01 *vs*. control, *n* = 7), but suppressed by TGF‐β (^#^ *P* < 0.01, ^##^ *P* < 0.01 *vs*. –TGF‐β, *n* = 7). All comparisons by two‐way ANOVA.

To see how the interaction between SrcFK inhibition and TGF‐β pre‐incubation on BK‐induced contraction relates to SrcFK expression and activity, we determined the effects of TGF‐β on c‐Src protein expression in hASMCs. We found that acute treatment with BK (30 s, 1 μm) enhanced SrcFK auto‐phosphorylation, but that this enhancement was not potentiated by prior exposure to TGF‐β and that pre‐exposure to TGF‐β reduced c‐Src protein content by ∼30% (Fig. 2 *A* and *B*). To account for the effect of altered c‐Src content on SrcFK phosphorylation, changes in auto‐phosphorylation were expressed in two ways: phospho/total, a proportional measure of the degree of phosphorylation (Fig. 2 *C*), and phospho/GAPDH, a measure of actual phosphorylated SrcFK content, relative to total cellular protein (Fig. 2 *D*). Without prior incubation with TGF‐β, acute exposure to BK produced a 2‐fold increase in SrcFK auto‐phosphorylation, while after TGF‐β pre‐incubation the enhancing effect of BK was significantly reduced. Basal SrcFK phosphorylation was also suppressed by TGF‐β, but only when expressed as phospho/GAPDH, linking it to the reduced c‐Src protein content.

### TGF‐β enhances ARHGEF1 expression, while BK triggers ARHGEF1 translocation

**Figure 3:**
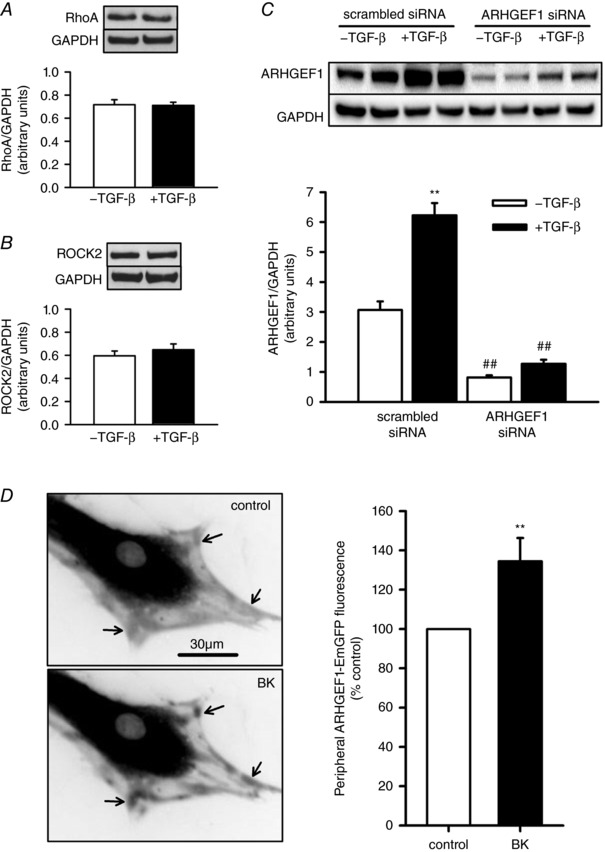
Effects of TGF‐β and BK on ARHGEF1 expression and translocation *A*–*C*, effects of TGF‐β incubation (10 ng ml^−1^, 24 h) on protein expression of RhoA (*A*, *n* = 6), Rho‐kinase (*B*, ROCK2, *n* = 5) and ARHGEF1 (*C*, after transfection with scrambled or ARHGEF1 siRNA, *n* = 9) in hASMCs, relative to GAPDH. ^**^ *P* < 0.01 *vs*. –TGF‐β, ^##^ *P* < 0.01 *vs*. scrambled siRNA, two‐way ANOVA. *D*, fluorescence imaging of live hASMCs transfected with ARHGEF1‐EmGFP. Arrows indicate peripheral regions in which ARHGEF1‐EmGFP concentrates when stimulated by addition of BK (1 μm). Data show relative changes in fluorescence in peripheral regions. ^**^ *P* < 0.01 *vs*. control, *n* = 11, paired *t* test.

Considering that the TGF‐β‐induced enhancement of contraction is dependent on RhoGEFs and Rho‐kinase, we next examined the effects of TGF‐β on expression, activity and translocation of RhoA, expression and activity of Rho‐kinase and the influence of the RhoGEF ARHGEF1 on those effects, in hASMCs. TGF‐β had no effect on protein expression of either RhoA or Rho‐kinase, relative to GAPDH (Fig. 3 *A* and *B*) but induced a doubling in protein content of ARHGEF1, relative to GAPDH (Fig. 3 *C*). In hASMCs transfected with EmGFP‐tagged ARHGEF1, fluorescence was concentrated in the perinuclear region, but with lower levels in the cell periphery. Addition of BK (1 μm) reversibly enhanced this fluorescence in distinct peripheral patches or spots (Fig. 3 *D*), indicating that ARHGEF1 translocates to these regions in response to stimulation by BK.

### TGF‐β influences RhoA activity and translocation: role of ARHGEF1

**Figure 4:**
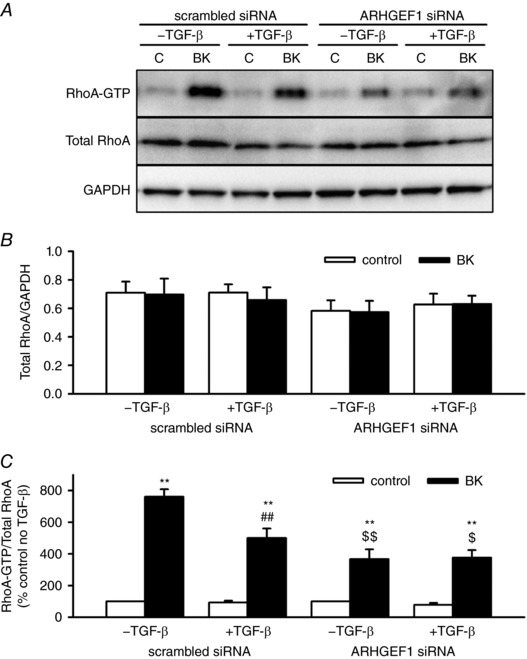
Effects of TGF‐β and ARHGEF1 siRNA on RhoA‐GTP content in hASMCs *A*, representative blots showing effects of acute BK treatment (1 μm, 30 s) with or without TGF‐β pre‐treatment (10 ng ml^−1^, 24 h) in hASMCs transfected with either ARHGEF1 siRNA or a scrambled siRNA control on RhoA‐GTP content, total RhoA or GAPDH as a loading control. *B*, data expressed as total/GAPDH show no overall effect of BK, TGF‐β or ARHGEF1 siRNA on total RhoA expression. *C*, data expressed as RhoA‐GTP/total show significant enhancement by BK (^**^ *P* < 0.01 *vs*. control) but partial suppression by TGF‐β (^##^ *P* < 0.01, *vs*. –TGF‐β) and ARHGEF1 siRNA (^$^ *P* < 0.05, ^$$^ *P* < 0.01 *vs*. scrambled siRNA). *n* = 7 for all data. All comparisons by three‐way ANOVA.

Although TGF‐β does not alter expression of RhoA or Rho‐kinase, it may alter their activity and/or cellular localization via the enhanced expression of ARHGEF1. Therefore, we next determined whether TGF‐β influences RhoA/Rho‐kinase activity by examining the effects of pre‐exposure to TGF‐β on RhoA‐GTP content and MYPT1 and MLC_20_ phosphorylation in hASMCs. We also examined the effects of TGF‐β on RhoA‐EmGFP translocation. To determine whether any of these changes were mediated via ARHGEF1, the effects of ARHGEF1 siRNA on these parameters were also investigated. Transfection of hASMCs with ARHGEF1 siRNA produced an approximately 75% reduction in ARHGEF1 protein expression compared to transfection with a scrambled siRNA (Fig. 3 *C*). Figure 4 shows the effects of BK, TGF‐β and ARHGEF1 siRNA on RhoA‐GTP content, a direct measure of total cellular RhoA activity. The positive control GTPγS consistently caused a several‐fold increase in RhoA‐GTP content relative to total RhoA (986 ± 69%, *n* = 7). As expected, BK also caused a several‐fold increase in RhoA activity and this increase was significantly inhibited in hASMCs transfected with an ARHGEF1 siRNA compared to a scrambled (control) siRNA (Fig. 4 *A* and *C*). The effect of BK was also partially suppressed by TGF‐β, but it was still further suppressed by the siRNA, resulting in no overall effect of TGF‐β on the BK‐induced response in hASMCs transfected with ARHGEF1 siRNA (Fig. 4 *A* and *C*). Neither TGF‐β nor ARHGEF1 siRNA affected RhoA‐GTP content in the absence of BK, and total RhoA content relative to GAPDH was not affected by any of the treatments used (Fig. 4 *B*).

**Figure 5:**
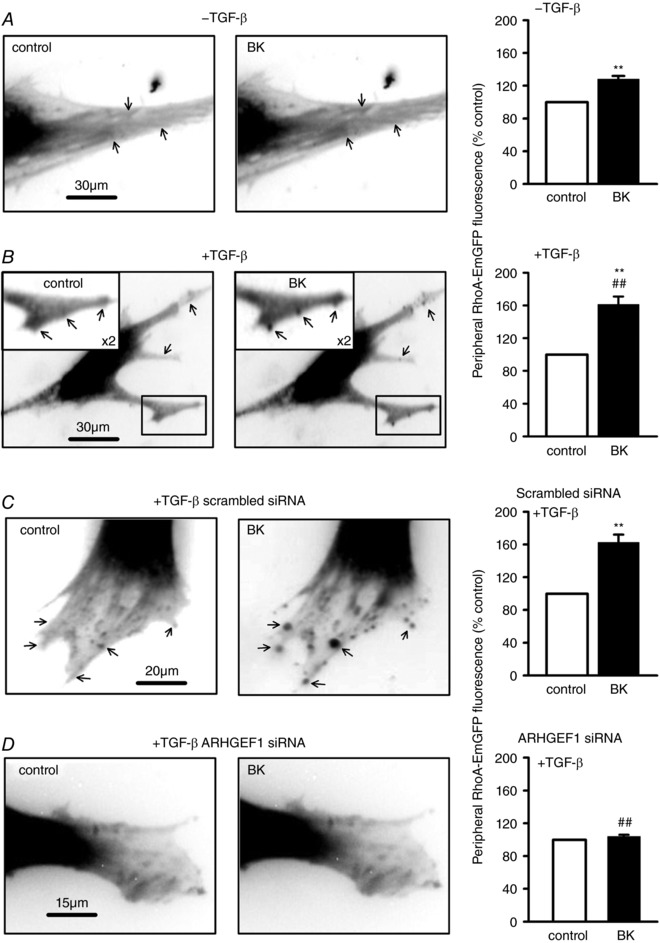
Effects of TGF‐β and ARHGEF1 siRNA on BK‐induced RhoA‐EmGFP translocation Fluorescence imaging of live hASMCs transfected with RhoA‐EmGFP. *A*, in the absence of TGF‐β pre‐incubation, addition of BK (1 μm) enhances peripheral fluorescence, as indicated by arrows. ^**^ *P* < 0.001 *vs*. control, *n* = 16. *B*, after TGF‐β pre‐incubation (10 ng ml^−1^, 24 h), response to BK is enhanced and is concentrated into distinct peripheral spots/patches, as indicated by arrows. ^**^ *P* < 0.001 *vs*. control, ^##^ *P* < 0.001 *vs*. –TGF‐β, *n* = 15. *C*, in hASMCs pre‐treated with TGF‐β, doubly transfected with RhoA‐EmGFP and scrambled siRNA, response to BK is similar to in *B*, as indicated by arrows. ^**^ *P* < 0.001 *vs*. control, *n* = 28. *D*, in hASMCs pre‐treated with TGF‐β, co‐transfected with RhoA‐EmGFP and ARHGEF1 siRNA, BK does not trigger translocation. ^##^ *P* < 0.001 *vs*. scrambled siRNA, *n* = 29. All comparisons by two‐way ANOVA.

In hASMCs transfected with RhoA‐EmGFP, application of BK induced a modest enhancement of fluorescence in the cell periphery (Fig. 5 *A*). After prior incubation with TGF‐β, this BK‐induced translocation was enhanced and was increasingly characterized by the appearance or intensification of spots/patches at the cell periphery or on cellular projections (Fig. 5 *B*). A similar pattern of BK‐induced RhoA‐EmGFP translocation was observed in hASMCs exposed to TGF‐β and transfected with scrambled siRNA (Fig. 5 *C*), while translocation was not observed in hASMCs exposed to TGF‐β and co‐transfected with ARHGEF1 siRNA (Fig. 5 *D*).

### TGF‐β enhances MYPT‐1/MLC_20_ expression and phosphorylation in hASMC: role of ARHGEF1

Considering that RhoA activates Rho‐kinase, which in turn enhances MLC_20_ phosphorylation via inhibition of MLCP, we next examined the effects of TGF‐β pre‐incubation and ARHGEF1 siRNA on protein expression and BK‐induced phosphorylation of MLC_20_ itself and of MYPT1, which when phosphorylated on Th696 results in MLCP inhibition.

**Figure 6:**
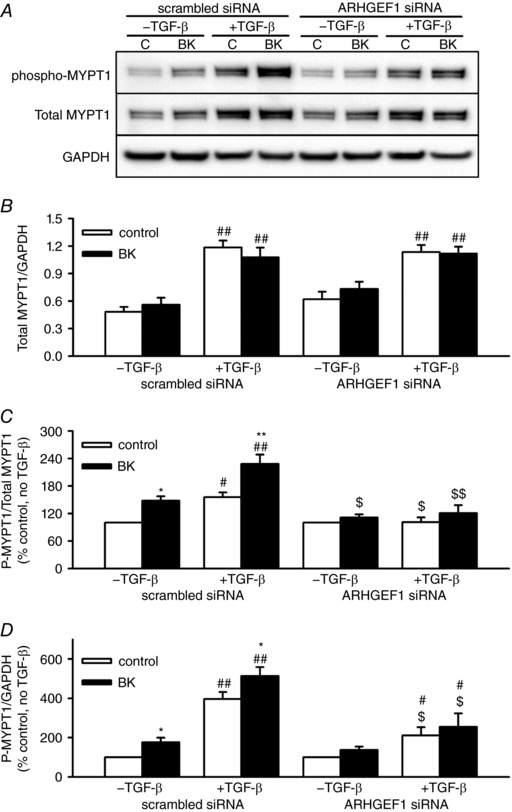
Effects of TGF‐β and ARHGEF1 siRNA on expression and BK‐induced phosphorylation of MYPT‐1 in hASMCs *A*, representative blots showing effects of acute BK treatment (1 μm, 30 s) with or without TGF‐β pre‐treatment (10 ng ml^−1^, 24 h) in hASMCs transfected with either ARHGEF1 siRNA, or a scrambled siRNA control, on phospho‐MYPT‐1 (Thr‐696), total MYPT1 or GAPDH as a loading control. *B*, data expressed as total/GAPDH show enhancement of MYPT1 protein expression by TGF‐β pre‐treatment, but no effect of ARHGEF1 siRNA or BK, ^##^ *P* < 0.01 *vs*. –TGF‐β. Data expressed as phospho/total (*C*) or phospho/GAPDH (*D*) show significant enhancement by both BK (^*^ *P* < 0.05, ^**^ *P* < 0.01 *vs*. control) and TGF‐β (^#^ *P* < 0.05, ^##^ *P* < 0.01 *vs*. –TGF‐β) and suppression by ARHGEF1 siRNA (^$^ *P* < 0.05, ^$$^ *P* < 0.01 *vs*. scrambled siRNA). *n* = 9 for all data. All comparisons by three‐way ANOVA.

Pre‐incubation with TGF‐β almost doubled the protein expression of MYPT1 in hASMCs transfected with the scrambled siRNA (relative to GAPDH). The ARHGEF1 siRNA did not alter this increase (Fig. 6 *A* and *B*). To account for changes in total MYPT1 content, we expressed changes in MYPT1 phosphorylation in two ways: relative to total MYPT1 (phospho/total, Fig. 6 *C*) and relative to total cellular protein (phospho/GAPDH, Fig. 6 *D*). In hASMCs transfected with scrambled siRNA and not pre‐incubated with TGF‐β, acutely applied BK enhanced MYPT1 phosphorylation. In hASMCs pre‐incubated with TGF‐β, both basal and BK‐induced MYPT1 phosphorylation was enhanced. This enhancement was greatest when expressed as phospho/GAPDH, but was still evident when expressed as phospho/total. In hASMCs transfected with ARHGEF1 siRNA, acute application of BK was unable to enhance MYPT1 phosphorylation, regardless of whether hASMCs were first incubated with TGF‐β or not. In hASMCs transfected with ARHGEF1 siRNA, TGF‐β still enhanced basal MYPT1 phosphorylation, but to a much lesser extent than in hASMCs transfected with scrambled siRNA. Furthermore, this residual enhancing effect of TGF‐β pre‐incubation was only evident when the data were expressed as phospho/GAPDH.

**Figure 7:**
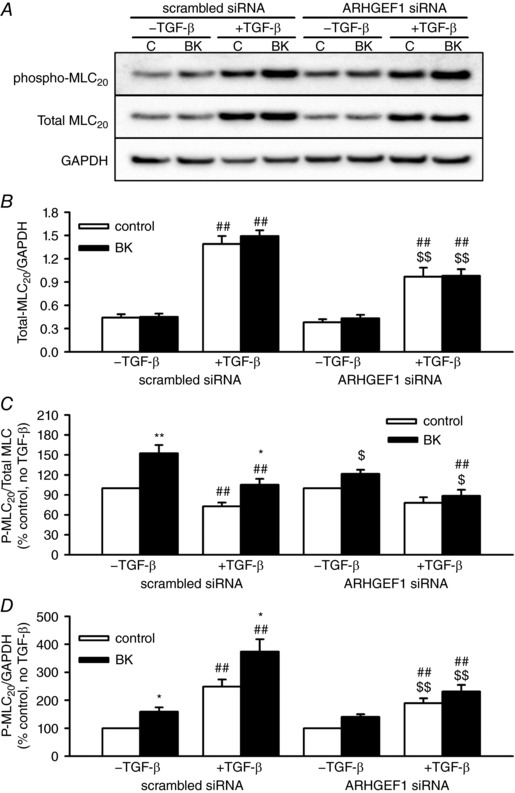
Effects of TGF‐β and ARHGEF1 siRNA on expression and BK‐induced phosphorylation of MLC_20_ in hASMCs *A*, representative blots showing effects of acute BK treatment (1 μm, 30 s) with or without TGF‐β pre‐treatment (10 ng ml^−1^, 24 h) in hASMCs transfected with either ARHGEF1 siRNA, or a scrambled siRNA control, on phospho‐MLC_20_ (Ser19), total MLC_20_ or GAPDH as a loading control. *B*, data expressed as total/GAPDH show enhancement of MLC_20_ protein expression by TGF‐β pre‐treatment (^##^ *P* < 0.01 *vs*. –TGF‐β), and partial suppression of this response by ARHGEF1 siRNA (^$$^ *P* < 0.01 *vs*. scrambled siRNA). Data expressed as phospho/total (*C*) or phospho/GAPDH (*D*) show significant enhancement by both BK (^*^ *P* < 0.05, ^**^ *P* < 0.01 *vs*. control), effects of TGF‐β pre‐treatment (^#^ *P* < 0.05, ^##^ *P* < 0.01 *vs*. –TGF‐β) and suppression by ARHGEF1 siRNA (^$^ *P* < 0.05, ^$$^ *P* < 0.01 *vs*. scrambled siRNA). *n* = 9 for all data. All comparisons by three‐way ANOVA.

In cells transfected with scrambled siRNA, MLC_20_ protein content was increased 3‐fold by TGF‐β (relative to GAPDH). Unlike with MYPT1, this increased protein expression was partially reversed in cells transfected with ARHGEF1 siRNA (Fig. 7 *A* and *B*). As with MYPT1, to account for TGF‐β‐induced MLC_20_ protein content, changes in MLC_20_ phosphorylation were expressed in two ways: relative to total MLC_20_ (phospho/total, Fig. 7 *C*) or relative to total cellular protein (phospho/GAPDH, Fig. 7 *D*).

In hASMCs transfected with scrambled siRNA and not pre‐incubated with TGF‐β, acutely applied BK enhanced MLC_20_ phosphorylation, as expected. After incubation with TGF‐β, BK still caused an increase in phosphorylation, but the overall effect of TGF‐β on basal and BK‐induced MLC_20_ phosphorylation depended on the way the data were expressed. When expressed as total/phospho, an approximately 30% suppressing effect of TGF‐β on phosphorylation was indicated, but when expressed as phospho/GAPDH, an approximately 2‐fold enhancing effect of TGF‐β on phosphorylation was indicated.

In hASMCs transfected with ARHGEF1 siRNA, acute application of BK was unable to significantly enhance MLC_20_ phosphorylation, regardless of whether hASMCs were first incubated with TGF‐β or not. This inhibitory action of ARHGEF1 siRNA was evident with either way the data were expressed, but was most evident when they were expressed as phospho/total (Fig. 7 *C*). Transfecting hASMCs with ARHGEF1 siRNA weakened the effects of TGF‐β on MLC_20_ phosphorylation compared to ASM transfected with scrambled siRNA: it prevented the apparent TGF‐β‐induced suppression when data were expressed as phospho/total and it suppressed the TGF‐β‐induced enhancement when data were expressed as phospho/GAPDH.

### BK and TGF‐β influence cofilin phosphorylation: role of ARHGEF1

**Figure 8:**
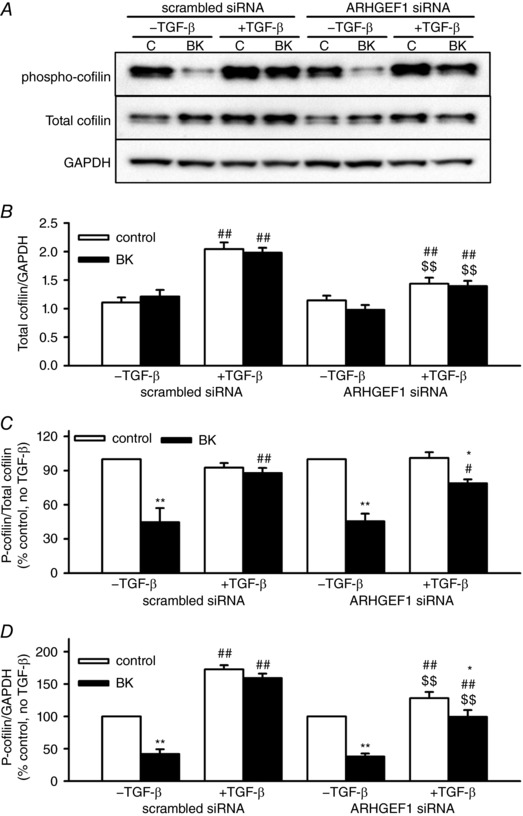
Effects of BK, TGF‐β and ARHGEF1 siRNA on cofilin expression and phosphorylation in hASMCs *A*, representative blots showing effects of acute BK treatment (1 μm, 30 s) with or without TGF‐β pre‐treatment (10 ng ml^−1^, 24 h) in hASMCs transfected with either ARHGEF1 siRNA, or a scrambled siRNA control, on phospho‐cofilin (Ser3), total cofilin or GAPDH as a loading control. *B*, data expressed as total/GAPDH show enhancement of cofilin protein expression by TGF‐β pre‐treatment (^##^ *P* < 0.01 *vs*. –TGF‐β), and partial suppression of this response by ARHGEF1 siRNA (^$$^ *P* < 0.01 *vs*. scrambled siRNA). Data expressed as phospho/total (*C*) or phospho/GAPDH (*D*) show significant de‐phosphorylation induced by BK (^**^ *P* < 0.01 *vs*. control), prevention of de‐phosphorylation/enhancement of basal phosphorylation by TGF‐β pre‐treatment (^#^ *P* < 0.05, ^##^ *P* < 0.01 *vs*. –TGF‐β) and partial suppression of the effects of TGF‐β by ARHGEF1 siRNA (^$$^ *P* < 0.01 *vs*. scrambled siRNA). *n* = 7 for all data. All comparisons by three‐way ANOVA.

In cells transfected with scrambled siRNA, cofilin protein content was increased approximately 2‐fold by TGF‐β (relative to GAPDH). This increased protein expression was partially reversed in cells transfected with ARHGEF1 siRNA (Fig. 8 *A* and *B*). As with MYPT1 and MLC_20_, basal and BK‐induced changes in cofilin phosphorylation were expressed in two ways: relative to total cofilin (phospho/total, Fig. 8 *C*) or relative to total cellular protein (phospho/GAPDH, Fig. 8 *D*). In hASMCs transfected with scrambled siRNA but not treated with TGF‐β, acute application of BK caused an approximately 50% de‐phosphorylation of cofilin, while in TGF‐β pre‐treated hASMCs, this BK‐induced de‐phosphorylation was prevented. In the absence of TGF‐β pre‐treatment, ARHGEF1 siRNA had no effect on the BK‐induced cofilin de‐phosphorylation, but in TGF‐β‐treated hASMCs the siRNA partially but significantly restored the BK‐induced cofilin de‐phosphorylation. When data were expressed as phospho/GAPDH, there was also a TGF‐β‐induced increase in total cofilin phosphorylation and a partial suppression of this effect by the ARHGEF1 siRNA (Fig. 8 *D*). These effects presumably relate to the influence of TGF‐β on total cofilin protein content.

**Figure 9:**
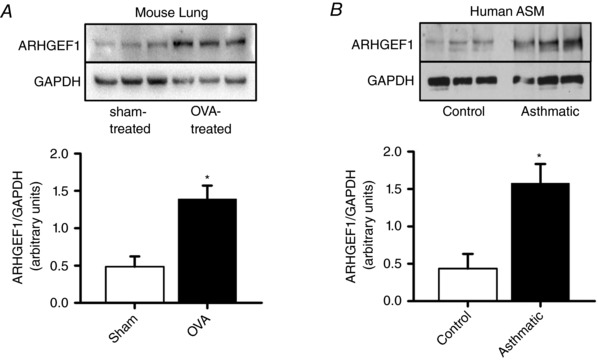
Effects of asthma and OVA‐sensitization on lung ARHGEF‐1 protein expression Protein expression of ARHGEF‐1 relative to GAPDH. Expression is enhanced by OVA‐sensitization in mouse lung (*A*, ^*^ *P* < 0.05 *vs*. sham‐treated, *n* = 3 in each group) and is greater in ASM of asthmatic subjects *vs*. healthy controls (*B*, ^*^ *P* < 0.05, *n* = 3 subjects in each group). All comparisons by unpaired *t* test.

Given the importance of ARHGEF1 in the above described effects of TGF‐β on RhoA/Rho‐kinase and cofilin, we also compared ARHGEF1 protein expression in healthy *vs*. asthmatic hASMCs and in lung of OVA‐sensitized *vs*. sham‐treated mice. ARHGEF1 expression was higher in hASMCs of asthmatic donors compared to healthy controls and was higher in lung of OVA‐sensitized mice compared to lung of sham‐treated controls (Fig. 9).

## Discussion

The results of this study show that TGF‐β enhances BK‐induced contraction in isolated rat bronchioles and enhances BK‐induced RhoA‐EmGFP translocation and MYPT‐1 and MLC_20_ phosphorylation in hASMCs, without potentiating BK‐induced enhancement of total cellular RhoA activity. Conversely, TGF‐β suppresses BK‐induced SrcFK auto‐phosphorylation and cofilin de‐phosphorylation. The effects of BK and TGF‐β on RhoA‐EmGFP translocation and activity and MYPT‐1/MLC_20_/cofilin phosphorylation are mediated in part by the guanine nucleotide exchange factor ARHGEF1.

RhoA/Rho‐kinase and SrcFK are all implicated in ASM responses to bronchoconstrictors (Chiba *et al*. 2001; Shaifta *et al*. 2015). Thus, both pathways have been suggested as potential novel therapeutic targets for asthma (Schaafsma *et al*. 2008; Katsumoto *et al*. 2013). We hypothesized that these signalling pathways may be altered by the inflammatory mediator TGF‐β, an important modulator of ASM phenotype, also implicated in asthma (McMillan *et al*. 2005). We found that, at concentrations similar to those detected in the airways of asthmatic patients (Redington *et al*. 1997; Torrego *et al*. 2007), pre‐incubation with TGF‐β enhanced BK‐induced contractile responses in isolated rat bronchioles. The BK concentration response was biphasic, as described previously (Shaifta *et al*. 2015), and TGF‐β selectively enhanced the amplitude of the low‐affinity component. Although TGF‐β reportedly induces hypertrophy of ASMCs (Halwani *et al*. 2011), an increased smooth muscle mass is unlikely to account for our observed effects of TGF‐β on BK‐induced contraction because KPSS‐induced contraction was unaffected by TGF‐β.

SrcFK inhibition with PP2 partially suppressed the bronchiole contractile response to BK and BK treatment enhanced SrcFK auto‐phosphorylation in hASMCs, as shown previously (Shaifta *et al*. 2015). Conversely, PP2 did not prevent the proportionate enhancing action of TGF‐β on contraction compared to media alone, and TGF‐β exerted a distinct inhibitory action on Src expression and activity. Thus, even though TGF‐β reportedly mediates some of its SMAD‐independent effects via activation of SrcFK (Samarakoon *et al*. 2008), our results suggest that although SrcFK may contribute to normal contractile responses in ASMCs, they do not contribute to the TGF‐β‐mediated enhancement of BK‐induced contraction. This is perhaps related to previous evidence that TGF‐β enhances SrcFK protein degradation as part of its anti‐proliferative action (Atfi *et al*. 1994; Fukuda *et al*. 1998).

We also previously showed that BK‐induced contraction in rat bronchioles is heavily dependent on Rho‐kinase activity and that BK enhances phosphorylation of the Rho‐kinase target MYPT1 in hASMCs, in line with that of MLC_20_ (Shaifta *et al*. 2015), suggesting a strong contribution of Rho‐kinase‐dependent MLCP inhibition to the contractile response (Somlyo & Somlyo, 2003). In the present study, the TGF‐β‐mediated enhancement of BK‐induced contraction was abolished by the Rho‐kinase inhibitor Y27632 and by Y16, an inhibitor of the RGS domain‐containing sub‐family of Rho‐specific guanine nucleotide exchange factors (ARHGEF1, ARHGEF11 and ARHGEF12) (Shang *et al*. 2013). Therefore, the effects of TGF‐β on contraction are likely to be mediated via enhanced expression or activity of components of the RhoGEF/RhoA/Rho‐kinase signalling pathway. Investigating this further, we found that TGF‐β had no effect on relative protein content of either RhoA or Rho‐kinase in hASMCs, but enhanced relative protein content of ARHGEF1, and downstream targets MYPT1, MLC_20_ as well as the actin severing protein cofilin. If protein content of RhoA and Rho‐kinase were unaltered by TGF‐β, then perhaps their activity is being enhanced via the TGF‐β‐mediated upregulation of ARHGEF1. We therefore examined RhoA activity directly (Rhotekin assay) and its subcellular translocation, which is often taken as a corollary of activity (but see below), together with activity of Rho‐kinase (MYPT1 phosphorylation) and MLC_20_ phosphorylation.

Measured using the Rhotekin assay, BK clearly causes activation of RhoA, as indicated by a several‐fold increase in total cellular RhoA‐GTP content relative to total RhoA. Significant suppression of this response by the ARHGEF1 siRNA confirms the importance of ARHGEF1 to RhoA activation by BK in hASMCs. Similarly, we found that BK triggered subcellular translocation of both ARHGEF1‐EmGFP and RhoA‐EmGFP to the periphery of live hASMCs and that the RhoA‐EmGFP translocation was abolished by co‐transfecting hASMCs with ARHGEF1 siRNA. TGF‐β also enhanced the BK‐induced RhoA‐EmGFP translocation, and this enhancement was abolished in hASMCs co‐transfected with an ARHGEF1 siRNA, suggesting a relationship between the effects of TGF‐β on ARHGEF1 expression and its effects on BK‐induced RhoA‐EmGFP translocation. Surprisingly, however, in hASMCs transfected with scrambled siRNA, TGF‐β did not enhance the BK‐induced increase in RhoA‐GTP content but partially suppressed it, while in cells transfected with ARHGEF1 siRNA, TGF‐β had no effect. Thus, following TGF‐β treatment, there is a dissociation between RhoA‐EmGFP translocation and RhoA activity. This is discussed further below in conjunction with the effects of BK, TGF‐β and ARHGEF1 siRNA on MYPT1 phosphorylation.

RhoA activates Rho‐kinase, and a good indicator of Rho‐kinase activity is the degree of phosphorylation of MYPT1, which when phosphorylated at Thr696 triggers inhibition of MLCP, resulting in a net enhancement of MLC_20_ phosphorylation at any given [Ca^2+^]_i_ (Feng *et al*. 1999). We therefore examined the effects of TGF‐β pre‐treatment and the ARHGEF1 siRNA on BK‐induced MYPT1 and MLC_20_ phosphorylation in hASMCs. Normally, protein phosphorylation data are expressed as a ratio of ‘phospho’ over ‘total’ for each protein (e.g. P‐MYPT1/MYPT1), to control for random variations in total content of each protein. However, when the treatment clearly also alters the expression of that protein, as is the case with our TGF‐β pre‐treatments, phospho‐protein content will be influenced by both the relative activity of the kinase responsible for the phosphorylation and the availability of the protein being phosphorylated. For this reason, phosphorylation data were expressed both as phospho/total, giving a measure of relative Rho‐kinase activity, and as phospho/GAPDH, giving a measure of actual phospho‐protein content relative to total cellular protein content. As expected, BK induced an increase in MYPT1 phosphorylation, while TGF‐β enhanced phosphorylation in both the absence and the presence of BK. This is broadly in line with its enhancing effect on RhoA‐EmGFP translocation but not with its partial suppressing effect on total RhoA‐GTP content. The relative increases in MYPT1 phosphorylation were evident regardless of the way the data were expressed, suggesting that the effect of TGF‐β was jointly due to increased Rho‐kinase activity and increased availability of MYPT1 for phosphorylation. The ARHGEF1 siRNA did not alter the TGF‐β‐mediated enhancement of MYPT1 protein content, but rendered the phosphorylation responses to BK statistically insignificant and greatly suppressed the enhancing effect of TGF‐β on this phosphorylation, especially when the data were expressed as phospho/total. These results provide clear evidence that ARHGEF1 is required for Rho‐kinase activity in hASMCs, in both the presence and the absence of TGF‐β.

It is unclear why TGF‐β enhances RhoA‐EmGFP translocation and Rho‐kinase activity but partially suppresses BK‐induced enhancement of total cellular RhoA‐GTP content. Translocation of native RhoA to the cell membrane/periphery as visualized by immunohistochemistry is often taken as a corollary of RhoGEF or GTPase activation (Chiba *et al*. 2004; Meyer *et al*. 2008) because it is assumed that translocation represents the movement of the RhoGEF to be activated by G_12/13_ or movement of RhoA to be activated by RhoGEFs. However, here we have examined the translocation of transfected RhoA‐EmGFP. The advantage of this method is that we were able to see the localization of the protein both in the absence and in the presence of BK in the same cell, but a potential disadvantage is that the attachment of GFP at the C‐terminal of RhoA may have interfered with prenylation of the protein and therefore its correct insertion into the cell membrane. Thus, the observed translocation may not be as direct a corollary of RhoA activity as it would otherwise be, although both are still dependent on ARHGEF1, albeit to varying degrees. Whatever the reason for the discrepancy between RhoA‐EmGFP translocation and RhoA activity, TGF‐β is clearly enhancing Rho‐kinase activity in ASM and this enhancement is dependent on ARHGEF1. The TGF‐β‐induced enhancement of ARHGEF1 expression could possibly result in a greater fraction of active RhoA being directed to parts of the cell where RhoA is able to activate Rho‐kinase, resulting in enhanced MYPT1 phosphorylation, despite an overall reduction in total cellular RhoA‐GTP. Alternatively, TGF‐β might induce a shift away from activation of Rho‐kinase by RhoA towards activation of Rho‐kinase by another small G‐protein, such as RhoB (Vasilaki *et al*. 2010), which has also been reported to be activated by ARHGEF1 in other cell types (Jaiswal *et al*. 2011). Considerable further work will be required to fully characterize the ways in which RhoGEF/RhoA/Rho‐kinase signalling complexes are influenced by TGF‐β in ASM and whether other Rho proteins also play a part.

Quantifying MLC_20_ phosphorylation reflects the balance between MLCK and MLCP activity as well as the total amount of MLC_20_ available for phosphorylation. TGF‐β induced a 3‐fold increase in relative MLC_20_ protein content. This has the potential to support a 3‐fold increase in cross‐bridge cycling and contraction, but for such an increase to occur there needs to be a similar fold shift in the balance between MLCK and MLCP activity. In the absence of TGF‐β or ARHGEF1 siRNA, BK enhanced MLC_20_ phosphorylation, as expected, but how this response was influenced by TGF‐β depended on the way the data were expressed. When expressed as phospho/total, TGF‐β appeared to partially suppress both basal and BK‐induced phosphorylation, but when expressed as phospho/GAPDH, it caused an approximate 2‐fold enhancement. A possible explanation for this discrepancy is that MLC_20_ protein expression was increased by a greater amount (3‐fold) than was total MLC_20_ phosphorylation (phospho/GAPDH, 2‐fold), resulting in an apparent reduction in relative phosphorylation (phospho/total) because not all of the extra MLC_20_ was being phosphorylated. Nevertheless, a 2‐fold increase in total MLC_20_ phosphorylation could support a similar increase in actin‐myosin cross‐bridge cycling, bearing in mind that TGF‐β also enhances expression of myosin heavy chain and α‐actin in ASMCs (Goldsmith *et al*. 2006; Oenema *et al*. 2012), and MLCK in other cell types (Rossi *et al*. 2011). By also examining the inhibitory effects of ARHGEF1 siRNA on MLC_20_ phosphorylation we are able to confirm that BK‐induced phosphorylation and the enhancing effect of TGF‐β were primarily due to increases in Rho‐kinase activity downstream of ARHGEF1, since the siRNA abolished the effects of TGF‐β on the phospho/total signal, which is proportional to relative Rho‐kinase activity. Interestingly, the TGF‐β‐induced enhancement of MLC_20_ protein expression was also partially dependent on ARHGEF1, supporting previous evidence for RhoA/Rho‐kinase contributing to TGF‐β‐induced contractile protein expression (Zhao *et al*. 2007; Tsapara *et al*. 2010; Ji *et al*. 2014). In retinal epithelium, TGF‐β enhances expression of another RhoGEF, GEF‐H1 (Tsapara *et al*. 2010), but to our knowledge this study provides the first evidence that ARHGEF1 is important in TGF‐β‐induced enhancement of both ASM contractile protein expression and cross‐bridge cycling.

Cofilin is a key mediator of cellular actin dynamics. By severing actin fibres and leaving barbed ends, it reduces the number of available contractile fibres, while on the other hand it increases the number of nucleation sites for the creation of new branched fibres (Albinsson *et al*. 2004; Andrianantoandro & Pollard, 2006; Leduc *et al*. 2016). In the absence of TGF‐β pre‐treatment, we found that BK triggered an approximately 50% de‐phosphorylation of cofilin, and that this effect was unaltered by the ARHGEF1 siRNA, suggesting no role for ARHGEF1, and therefore potentially no role for RhoA/Rho‐kinase, in the regulation of cofilin activity (Zhang *et al*. 2015). These results are consistent with a previous report in tracheal smooth muscle, where acetylcholine activates the phosphatase PP2B, which de‐phosphorylates and activates cofilin (Zhao *et al*. 2008). As they suggest, this makes sense if cofilin provides a pool of G actin from which new contractile fibres and their attachments can be made (Zhao *et al*. 2008), rather than causing a net reduction in actin polymerization, as reportedly occurs in other smooth muscles (Zeidan *et al*. 2007; Dai *et al*. 2008). Interestingly, after pre‐incubation with TGF‐β,  BK no longer induced de‐phosphorylation of cofilin, while at the same time cofilin expression was enhanced. Considering that TGF‐β also reportedly enhances actin polymerization in lung slices (Oenema *et al*. 2013), an alternative interpretation of the role of cofilin is that bronchoconstrictors could be activating PP2B and cofilin as a ‘braking action’ against their MLCK/Rho‐kinase‐dependent pro‐contractile actions (Vardouli *et al*. 2005; Sandbo *et al*. 2011). Thus TGF‐β could potentially be removing the ‘brake’ by causing a shift in favour of cofilin phosphorylation and inhibition of its actin severing activity. However, regardless of whether these effects of TGF‐β on cofilin are pro‐contractile or otherwise, they are only modestly influenced by ARHGEF1. The effects of the siRNA are to partially reverse the TGF‐β‐induced enhancement of cofilin expression and to only partially restore the BK‐induced cofilin de‐phosphorylation. Thus, ARHGEF1 is likely to be of minimal importance to TGF‐β‐mediated effects on actin polymerization.

**Figure 10:**
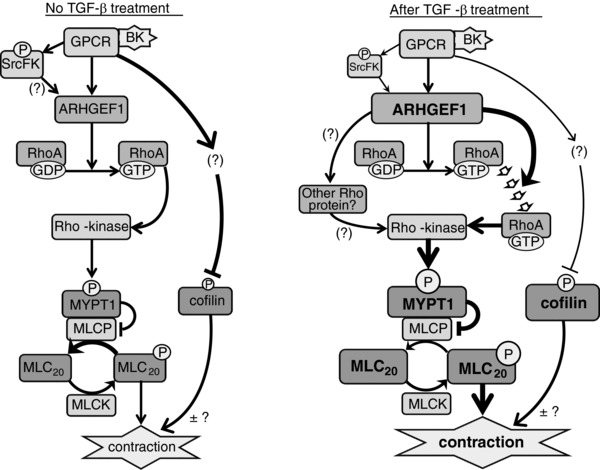
Role of ARHGEF1, SrcFK, RhoA, Rho‐kinase and cofilin in BK‐induced contraction in ASM and the influence of TGF‐β on their relative contributions *Without TGF‐β treatment*. It is established that G‐protein coupled receptor (GPCR)‐induced ASM contraction is dependent on sequential activation of RhoA and Rho‐kinase, resulting in MYPT1 phosphorylation, MLCP inhibition and enhanced MLC_20_ phosphorylation. We show that BK also induces SrcFK activation and translocation of ARHGEF1. RhoA translocation and activation, MYPT1 and MLC_20_ phosphorylation are all partially ARHGEF1‐dependent. Also, contraction is partially SrcFK‐, RhoGEF‐ and Rho‐kinase‐dependent. SrcFK possibly act upstream of ARHGEF1 (but not tested in this study). BK inhibits cofilin phosphorylation, but how this influences contraction remains to be determined. *After TGF‐β treatment*. Expression and BK‐induced activity of c‐Src are suppressed, while expression of ARHGEF1, MYPT1, MLC_20_ and cofilin are enhanced. BK‐induced RhoA activity is partially suppressed, while RhoA translocation, MYPT‐1 phosphorylation, MLC_20_ phosphorylation and contraction are all enhanced. Enhancement of RhoA translocation and MYPT1/MLC_20_ phosphorylation are partially ARHGEF1‐dependent, while enhanced contraction is Rho‐kinase and RhoGEF‐dependent. Enhanced Rho‐kinase activity may result from enhanced site‐specific RhoA translocation, to counter the reduced total RhoA activity, or from activation of another Rho‐protein (both untested in this study). Cofilin phosphorylation is enhanced, but is largely independent of ARHGEF1 and whether this contributes to enhanced contraction remains to be determined. Key: size of text/box reflects degree of protein expression. Size of ‘P’ reflects degree of phosphorylation. Thickness of lines/arrows reflects strength of effect. The row of block arrows represents translocation. (?) or (±?) denotes ‘not tested in this study’.

In summary, our results show that TGF‐β enhances Rho‐kinase activity and Rho‐kinase‐dependent contraction in ASM. Furthermore, ARHGEF1, a Rho‐specific guanine nucleotide exchange factor, is an important mediator of both the normal BK‐induced responses and their enhancement by TGF‐β (summarized in Fig. 10). In light of these findings and previous evidence showing the contribution of TGF‐β to airway remodelling and hyper‐contractility (Redington *et al*. 1997; Matsunaga *et al*. 2006; Torrego *et al*. 2007), we also show that ARHGEF1 is itself upregulated in hASMCs of asthmatic donors compared to healthy controls and is enhanced in lung of OVA‐sensitized mice compared to sham‐treated controls. Although a direct link between ARHGEF1, enhanced smooth muscle Rho‐kinase activity and abnormal airway function in asthmatic airways remains to be determined, our study supports previous work implicating ARHGEF1 in asthmatic T lymphocyte cytokine production (Brown *et al*. 2007), making it a prime candidate for pharmacological intervention in airway responsiveness and asthma.

## Additional information

### Competing interests

None.

### Author contributions

Intellectual content and study design: GK, JW, YS, CM, NI. Collection, analysis and interpretation of data: YS, CM, NI, KO, DW, GK. Drafting manuscript and graphical representation of data: GK. Critical evaluation of manuscript: JW, YS, CM. All authors approved the submission of this version.

### Funding

Wellcome Trust: #087776. British Heart Foundation: FS/12/43/29608
